# SARS-CoV-2 Omicron RBD shows weaker binding affinity than the currently dominant Delta variant to human ACE2

**DOI:** 10.1038/s41392-021-00863-2

**Published:** 2022-01-05

**Authors:** Leyun Wu, Liping Zhou, Mengxia Mo, Tingting Liu, Chengkun Wu, Chunye Gong, Kai Lu, Likun Gong, Weiliang Zhu, Zhijian Xu

**Affiliations:** 1 CAS Key Laboratory of Receptor Research, Drug Discovery and Design Center, Shanghai Institute of Materia Medica, Chinese Academy of Sciences, Shanghai, 201203 China; 2 College of Computer Science and Technology, National University of Defense Technology, Changsha, 410073 China; 3 Center for Drug Safety Evaluation and Research, Shanghai Institute of Materia Medica, Chinese Academy of Sciences, Shanghai, 201203 China

**Keywords:** Computational biology and bioinformatics, Biochemistry

**Dear Editor**,

SARS-coronavirus-2 (SARS-CoV-2) Omicron variant (B.1.1.529) is of great concern to the world due to its multiple mutations that may have an impact on transmissibility and immune evasion.^1^ Compared to the wild type (WT), Omicron carries as many as 30 single point mutations, 3 deletion mutation and one insertion mutation on its spike protein. Strikingly, there are 15 mutations observed in the Omicron receptor-binding domain (RBD), 10 of which are in the receptor-binding motif (RBM) that human angiotensin-converting enzyme 2 (ACE2) and most monoclonal antibodies (mAbs) interact directly with. As a comparison, the currently dominant variant Delta (B.1.617.2) has only 2 mutations (L452R and T478K) in its RBM and additional K417N and E484K mutations sometimes. Therefore, Omicron variant may significantly impact the binding affinity to ACE2 and effectiveness of currently available mAbs. Consequently, Omicron mutant has aroused wide concern, many countries have taken measures on entry restrictions to prevent its rapid spread. However, the transmissibility and immune evasion risk of Omicron have not been properly evaluated.

The spike protein of WT SARS-CoV-2 has 1273 amino acids (UniProt ID: P0DTC2), and its RBD is composed of residues 319–541 and RBM is of residues 437–507.^2^ The currently dominant Delta variant has only 4 mutations on its RBD (RBD_Delta_), much less than that on the Omicron RBD (RBD_Omicron_) (Fig. 1a). It could be seen that the 15 mutations of RBD_Omicron_ are not evenly distributed in RBD, but rather crowed in its RBM with 10 residues, *viz*., N440K, G446S, S477N, T478K, E484A, Q493K, G496S, Q498R, N501Y and Y505H (Fig. 1a). By checking the effect of single mutation on ACE2 binding affinity reported by Bloom et al.^3^, it was found that 9 RBD_Omicron_ mutations (S371L, S373P, S375F, K417N, G446S, E484A, G496S, Q498R, Y505H) should decrease the binding affinity to ACE2 while the other 6 mutations (G339D, N440K, S477N, T478K, Q493K, N501Y) should increase the binding affinity, resulting in a challenge of predicting its transmissibility and potential immune evasion risk. Accordingly, molecular dynamics (MD) simulations and ELISA bioassay were employed in this letter to study the binding affinity between WT/Delta/Omicron RBDs and ACE2/mAbs. The details of the methods, RMSDs, structures, and movie are presented in the Supplementary Materials and Methods, Tables S1, S2, Figs. S1–S3 and Movie S1.

**Fig. 1:**
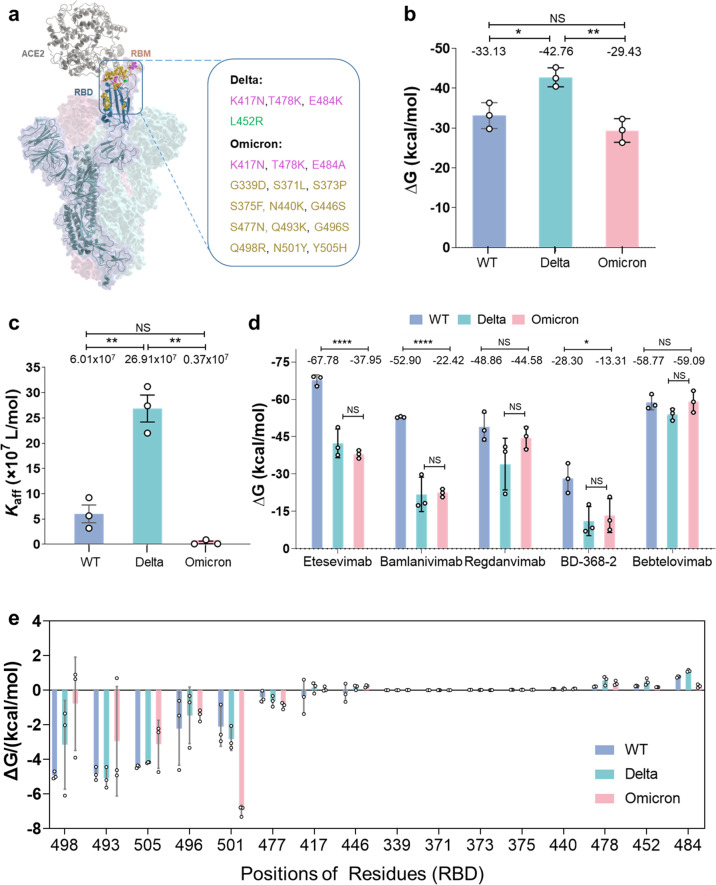
Binding affinity of ACE2 to the RBDs of WT, Delta and Omicron variants. **a** Mapping SARS-CoV-2 RBD mutations on the three-dimensional structure. The spike protein trimer is shown as surface. Identical mutations sites in both Delta and Omicron variants are shown as magenta. Other RBD mutations in Delta and Omicron variants are shown as green and yellow, respectively. **b** The predicted ACE2-RBD binding free energy (kcal/mol) of WT, Delta and Omicron variants. **c** RBD-ACE2 affinity constants measured by ELISA. **d** The predicted mAb-RBD binding free energy (kcal/mol) of WT, Delta and Omicron variants. **e** Energy contributions of 16 RBD mutations in Delta or Omicron variants. NS, not significant; *P < 0.05, **P < 0.01, ***P < 0.001, ****P < 0.0001

Computationally, we performed 200 ns all-atom MD simulations with the software Gromacs2020.2 at 300 K for WT, Delta and Omicron variant RBDs-ACE2 complexes in triplicate (600 ns in total for each system, Supplementary Figs. S1–S3), respectively, and calculated the binding free energy (Δ*G*) by MM/GBSA method with the software Amber18. The predicted Δ*G* of RBD_Omicron_ to ACE2 is −29.43 ± 3.01, and that of RBD_Delta_ is −42.76 ± 2.38 kcal/mol (*P* = 0.0039) (Fig. 1b). Notably, RBD_Omicron_ has comparable binding free energy in comparison with WT RBD (RBD_WT_) to ACE2 (−33.13 ± 3.26 kcal/mol, *P* = 0.22). Therefore, the results would suggest that Omicron variant should have similar infection capability to that of WT virus but milder than Delta, if the capability is mainly determined by RBD-ACE2 binding affinity.

Experimentally, we measured the affinity constant (*K*_*aff*_) of human ACE2 to RBD_WT_, RBD_Delta_ and RBD_Omicron_, respectively, by using non-competitive ELISA approach. The determined *K*_*aff*_ values of ACE2-RBD_WT_, ACE2-RBD_Delta_ and ACE2-RBD_Omicron_ are 6.01 ± 3.02 × 10^7^, 26.91 ± 0.46 × 10^7^ and 0.37 ± 4.66 × 10^7^ L/mol, respectively (Fig. 1c, Supplementary Fig. S4). Statistically, Delta variant is significantly stronger than WT and Omicron, while there is no significant difference between WT and Omicron. The results are in good agreement with the observation from MD simulations.

To evaluate the potential immune evasion risk by the Omicron variant, the binding affinity between 5 mAbs and WT/Delta/Omicron RBDs were calculated by MM/GBSA approach based on MD simulations in triplicate (Fig. 1d and Supplementary Table S3), of which the former three are launched. Remarkably, RBD_Omicron_ has much weaker binding affinity to the two launched mAbs Etesevimab (Δ*G* = −37.95 ± 1.63 kcal/mol) and Bamlanivimab (−22.42 ± 1.61 kcal/mol) than WT (−67.78 ± 2.12 and −52.90 ± 0.29 kcal/mol, respectively), indicating a high risk of immune evasion of Omicron to the two launched mAbs. Another marketed mAb, Regdanvimab, has a weak trend of decreased binding affinity but without significance to either WT or Delta. The newly reported experimental data reveal that the neutralization capability of the 3 marketed mAbs to RBD_Omicron_ decrease by 3 orders of magnitude, which is in agreement with our prediction in general (details shown in Supplementary Text).^4^ Impressively, Bebtelovimab in clinic study has strong affinity to either WT or Delta and Omicron variants, indicating this mAb may have high neutralization efficacy to WT and the 2 variants. The very weak RBD binding affinity of BD-368-2, a mAb in clinic trial, to both variants suggests its poor neutralization efficacy. Therefore, the new variant Omicron may have high immune evasion risk to most of the existing mAbs.

However, we should point out that the weaker RBD_Omicron_ binding to the ACE2 does not certainly mean a lower transmissibility as it may be affected by many different signal pathways and factors throughout the whole cycle of virus infection and replication, and the spike trimer may function differently from a single RBD.^5^ Accordingly, we like to point out that close attention should be paid intensively on the Omicron variant even if its ACE2 binding affinity becomes lower than Delta, as its high immune evasion risk from most of the existing mAbs may possibly enable its easy transmission among the vaccinated people.

For exploring the details of the interaction mechanism between the RBDs and the ACE2 at molecular and atomic levels, the binding free energies were decomposed onto every mutated residue based on the MD trajectories. It was found that the energy contribution of the residues 498, 493, 505 and 496 in RBD_Omicron_ are weaker than that in WT (Fig. 1e), while that of Y501 in Omicron is greatly stronger than N501 in WT. The opposite effects make the change of interaction between RBD_Omicron_ and ACE2 very complicated, but finally resulting in a similar binding affinity of RBD_Omicron_-ACE2 to that of RBD_WT_-ACE2. The detailed interaction mechanism and contributions are summarized in Supplementary Figs. S5 and S7 and discussed in. In overall, it is a combinatorial effect of the multiple mutations of Omicron on its similar binding free energy to RBD_WT_, while the strong binding free energies of RBD_Delta_ to ACE2 could be attributed to the hydrophobic interaction from the residues of F486, Y489 and F490 (Supplementary Fig. S8).

In conclusion, both MD simulation and ELISA bioassay results suggest that Omicron variant possesses comparable binding affinity to human ACE2 in comparison with the wild type SARS-CoV-2, but much weaker binding affinity than Delta variant. Accordingly, Omicron variant might have similar infection capability to that of WT virus but milder than Delta if the capability depends mainly on the binding affinity. In addition, the MD simulations indicate that the new variant Omicron has high risk of immune evasion. Accordingly, close attention should be paid intensively to Omicron as its high immune evasion risk may possibly enable its easy transmission.

## Supplementary information

Supplementary Materials

Movie S1 The interaction modes between ACE2 and 3 different RBDs

## Data Availability

The initial pdb structures and parameter files in Gromacs format can be downloaded from https://github.com/Zhijian-Xu/Omicron_RBD_MD and other data that support the findings of this study are available from the corresponding authors upon reasonable request.
