# Supplementary material for: SARS-CoV-2 Omicron RBD shows weaker binding affinity than the currently dominant Delta variant to human ACE2
Source: Signal Transduct Target Ther. 2022 Jan 5;7:8. doi: 10.1038/s41392-021-00863-2 (PMC8727475; doi:10.1038/s41392-021-00863-2)
Supplement: Supplementary file 1 — Supplementary Materials [file 41392_2021_863_MOESM1_ESM.pdf]

Supplementary Materials for

SARS-CoV-2 Omicron RBD shows weaker binding affinity than the currently dominant Delta variant to human ACE2

Leyun Wu[1, #], Liping Zhou[1, #], Mengxia Mo[2,#], Tingting Liu[3,#], Chengkun Wu[2,#], Chunye Gong[2], Kai Lu[2], Likun Gong[3, *], Weiliang Zhu[1, *], and Zhijian Xu[1, *]

[1]CAS Key Laboratory of Receptor Research; Drug Discovery and Design Center, Shanghai Institute of Materia Medica, Chinese Academy of Sciences, Shanghai 201203, China

[2]College of Computer Science and Technology, National University of Defense Technology, Changsha 410073, China

[3]Center for Drug Safety Evaluation and Research, Shanghai Institute of Materia Medica, Chinese Academy of Sciences, Shanghai 201203, China

[#]Leyun Wu, Liping Zhou, Mengxia Mo, Tingting Liu, and Chengkun Wu contributed equally to this work.

[*]Correspondence to: lkgong@cdser.simm.ac.cn (L.G.), wlzhu@simm.ac.cn (W.Z.), zjxu@simm.ac.cn (Z.X.).

This PDF file includes:

Materials and Methods
Supplementary Text
Figures S1 to S8
Table S1 to S3
Captions for Movie S1

Materials and Methods

Preparation of the three dimensional structures of mAb-S protein and ACE2-S protein complexes

The Omicron spike trimer was modelled by SWISS-MODEL Server in Alignment mode.[1] The Omicron homology model with the RBD up was chosen for further analysis. The Omicron spike were superimposed to a spike/ACE2 complex (PDB ID: 6VW1[2]) to create an Omicron-ACE2 complex structure. For the RBD-ACE2 system, zinc ion in the structure was retained. We retrieved 5 structures of marketed or clinical RBD-specific antibodies bound to S protein from the Protein Data Bank. The Omicron RBD domain containing residue 334-526 were truncated from the full-length S protein. In order to get the intact structures for WT/Delta RBDs and antibodies, missing residues in flexible loops were modeled using SWISS-MODEL. Delta RBD model was created by PyMOL2.5[3] to yield K417N and E484K on the basis of 7VVS.[4] The Delta model in our simulations have 4 mutations: K417N, L452R, T478K, and E484K.

System preparation

Protonation states were assessed using H++ 3.2[5,6] at pH 7.4. A cubic explicit water box described using the TIP3P model was used to solvated the complex system, which was extended by 10 Å from the solute. An atmosphere of 150 mM NaCl was also included in all simulations. The generated models were parametrized using amber ff14SB force fields[7] for protein. Subsequently, the parameter files created by tleap in Amber18[8] were converted to gromacs format. 5000 steps of minimization including 2500 steps of steepest descent minimization and 2500 steps of conjugate gradient minimization were performed to remove bad contacts during the energy minimization phase. Equilibration in NPT ensemble was run at 1.0 bar and 300 K for 500 000 steps at 2 fs/step. Gromacs2020.2[9,10] software package was used to run the minimization, equilibration simulations with position constraints (1 kcal/mol/Å^2) on protein.

Molecular dynamics (MD) simulations

Mdrun module in Gromacs2020.2 was used to perform 200ns MD production

simulations in triplicate at 300 K, 1 bar for all complexes. Temperature and pressure were controlled by Langevin thermostat[11] and a Nosé-Hoover Langevin barostat.[12,13] Bonds involving hydrogen atoms were fixed by the SHAKE algorithm.[14] The cutoff distance applied for van der Waals interactions is 10 Å. In order to deal with the coordination effects between zinc and adjacent nitrogen atom of HIS or oxygen atom of GLU in the RBD-ACE2 systems, simple distance restraint method was used. The force constant for distance restraints and time constant for distance restraints running average were set to 1.0 kJ/mol/nm^2 and 0.0 picosecond, respectively. All simulations were performed using particle-mesh Ewald (PME) for long-range electrostatic interactions.[15] Mdconvert[16] was used to convert the trajectories to amber format. Cpptraj module in Amber18 was used for trajectory processing and analysis.

Binding free energy calculation

Binding free energy (ΔG) of RBD-antibody or ACE2-RBD complexes was calculated by MM/GBSA[17] method using GB OBC model (igb = 5) with a salt concentration of 150 mM. 750 snapshots evenly extracted from 50-200ns trajectories were used for binding free energy calculation. In this study, the internal and external dielectric constants were set to be 1.0 and 78.5 separately. The free energy decomposition analysis was carried out using an internal program with idecomp = 1.

Binding ELISA

To measure the affinity constant of SARS-CoV-2 Spike protein RBD_{WT} (His Tag) (GenScript), SARS-CoV-2 Spike RBD_{Delta} (K417N, L452R, T478K) protein (His Tag) (Sino Biological), and SARS-CoV-2 B.1.1.529 (Omicron) Spike RBD protein (His Tag) (Sino Biological) against the ACE2-Fc protein (GenScript), a non-competitive ELISA was performed.[18] After being coated with 1, 2, and 4 μg/mL ACE2-Fc Tag protein overnight at 4 ℃, the 96-well plates were washed with 0.1% PBST, blocked with 3% BSA in PBS (Thermo Fisher Scientific, Waltham, MA, USA), and then incubated with 4- fold serial dilutions of RBDs at 37 ℃ for 1 h. The tested concentrations were between

10 μg/mL and 38 pg/mL. Thereafter, His tag antibody (HRP) (Sino Biological) was added at 1:10000 dilution, and the plate was incubated at 37 ℃ for 1 h. After washing the plate thrice with 0.1% PBST, TMB substrate (SeraCare, Milford, MA, USA) was added and the reaction was stopped with 2 M H_2SO_4, and the absorbance was read at 450 nm with Infinite F50 microplate reader (Tecan Trading AG, Zürich, Switzerland). The following formula for calculation of affinity constant (K_{aff}) in 1/mol (M^{-1}) was used:

$$K_{aff} = \frac{(n-1)}{2(n[Ab]1 - [Ab]2)}$$

where n represents the ratio between the highest and the lowest ACE2 concentration for the three possible comparisons. In a comparison between two ACE2 concentrations, [Ab]1 represents the molar RBD concentration calculated for OD-50 (half of maximum OD450 nm), corresponding to the lower ACE2 concentration. [Ab]2 represents the molar RBD concentration calculated for OD-50 measured at 450 nm, corresponding to the higher ACE2 concentration. The calculation of [Ab]1 and [Ab]2 was carried out by interpolating the value of OD-50 in the curve of OD450 nm vs. RBD concentration, fitting the curve to a four-parameters logistic regression by GraphPad Prism version 9.1.1 (GraphPad Software, San Diego, California, USA). The K_{aff} value for each RBD represents the mean ± the standard deviation (SD) of the three calculated K_{aff} values.

The ACE2 binding affinities of different RBDs by the ELISA method in this letter (K_{aff}=6.01±3.02×10^7 L/mol for RBD_{WT}, 26.91±0.46×10^7 L/mol for RBD_{Delta}) show the same decrease tendency to those determined by either SPR method with Biacore 3000 (K_D of RBD_{WT} = 8.3 nM, K_D of RBD_{Delta} = 4.0 nM)[19] (RBD_{Delta} is stronger than RBD_{WT}) or a BLI based method with Octet RED96E (Sartorius) (K_D of RBD_{WT} = 21.3 nM).[20] In addition, the ELISA assay in this study is performed in same plate under the same assay condition, leading to lower variations and also high throughput capability. Furthermore, in comparison with other methods, such as SPR and BLI based methods, the ELISA method does not need to further derivatize the RBD molecules to be analyzed, which avoids the potential effect of derivatization on the affinity measurement of the molecules. Therefore, ELISA is employed for this study.

Statistics analysis

Homogeneity of variance was tested by F test. Subsequently, in order to analyze

whether there is significant difference between the binding affinities for either wild type, Delta or Omicron RBD to ACE2 or mAbs, the two-tailed unpaired Student's t-test with equal or unequal variance was used for every two groups. *P* values of less than 0.05 were considered to be significant.

Supplementary Text

Effects of Omicron variant on the binding affinities to three marketed mAbs

We performed a comprehensive literature survey and found that the experimentally determined binding affinity of all the 3 marketed mAbs (Etesevimab, Bamlanivimab and Regdanvimab) calculated in this letter to the Omicron RBD were reported in the literature reported on 14-12-2021,[21] which indicate that all the binding affinities of the 3 mAbs to $RBD_{Omicron}$ decrease by 3 orders of magnitude in comparison with that of RBD_{WT}. Our calculations show that $RBD_{Omicron}$ has much weaker binding affinities to the 2 marketed mAbs (Etesevimab and Bamlanivimab, ΔG=-37.95 and -22.42 kcal/mol) in comparison with the RBD_{WT} (-67.78 and -52.90 kcal/mol), demonstrating a good agreement with the experimental results of high immune evasion risk. The calculated binding affinity of $RBD_{Omicron}$ to Regdanvimab (-44.58 kcal/mol) also shows a decrease trend of binding affinity compared to RBD_{WT} (-48.86 kcal/mol) but without significance.

In addition, Xie and Ho reported their bioassay results of the binding affinity of $RBD_{Omicron}$ to the 2 marketed mAbs (Etesevimab and Bamlanivimab),[22,23] also shows a good agreement with our prediction. In overall, the prediction in this letter is in agreement with conclusion from bioassay.

Interaction between the RBDs and the ACE2 at molecular and atomic level

The binding free energy decomposition of residues (Fig. 1e) shows that energy contribution of N501 in RBD_{WT} is -2.12 $\pm$1.13 kcal/mol, while the energy contribution of Y501 in $RBD_{Omicron}$ is -6.97 $\pm$0.30 kcal/mol (a stronger attraction). By analyzing molecular dynamic trajectories, we found that $Y501_{RBD}$ and $Y41_{ACE2}$ could form a

strong π-π stacking interaction (Figure S5a). The center of mass distance between $Y501_{RBD}$ and $Y41_{ACE2}$ is around 5.5 Å throughout the 200ns trajectory (Figure S5b).

The common feature of Q493K and Q498R mutation is that both of them mutates from electrically neutral Q to positively charged amino acids (K and R). By binding free energy decomposition, the energy contributions of both K493 and R498 are decreased. Specifically, the energy contribution of K493 in $RBD_{Omicron}$ decreased about 1.90 kcal/mol to Q493 in RBD_{WT}, and the energy contribution of R498 in $RBD_{Omicron}$ decreased about 4.15 kcal/mol to Q498 in RBD_{WT}. Q493 in RBD_{WT} could form tight interactions with K31 and E35 in ACE2 (Figure S6). By calculating distances of $K31_{ACE2}$-$Q493_{RBD}$ and $K31_{ACE2}$-$K493_{RBD}$ (Figure S6a), we found that $K31_{ACE2}$ moves away from $K493_{RBD}$, which may be due to the repulsion of lysine with the same electrical properties. $K353_{ACE2}$ also moves away from $R498_{RBD}$ (Figure S7c). Hence, we speculate that the repulsion between positively charged residues is the main cause of the decreased binding free energy in ACE2-$RBD_{Omicron}$. $K493_{RBD}$ forms a tighter interaction with $E35_{ACE2}$ (Figure S6b), which partly compensates for the effect of positively charge repulsion. While the lack of compensation effect in $R498_{RBD}$ (Figure S7a & S7b) leads to its severe decreased interactions with ACE2.

We also performed the binding free energy decomposition for all the residues on different RBMs to human ACE2, which is depicted in Figure S8.

Figure S1

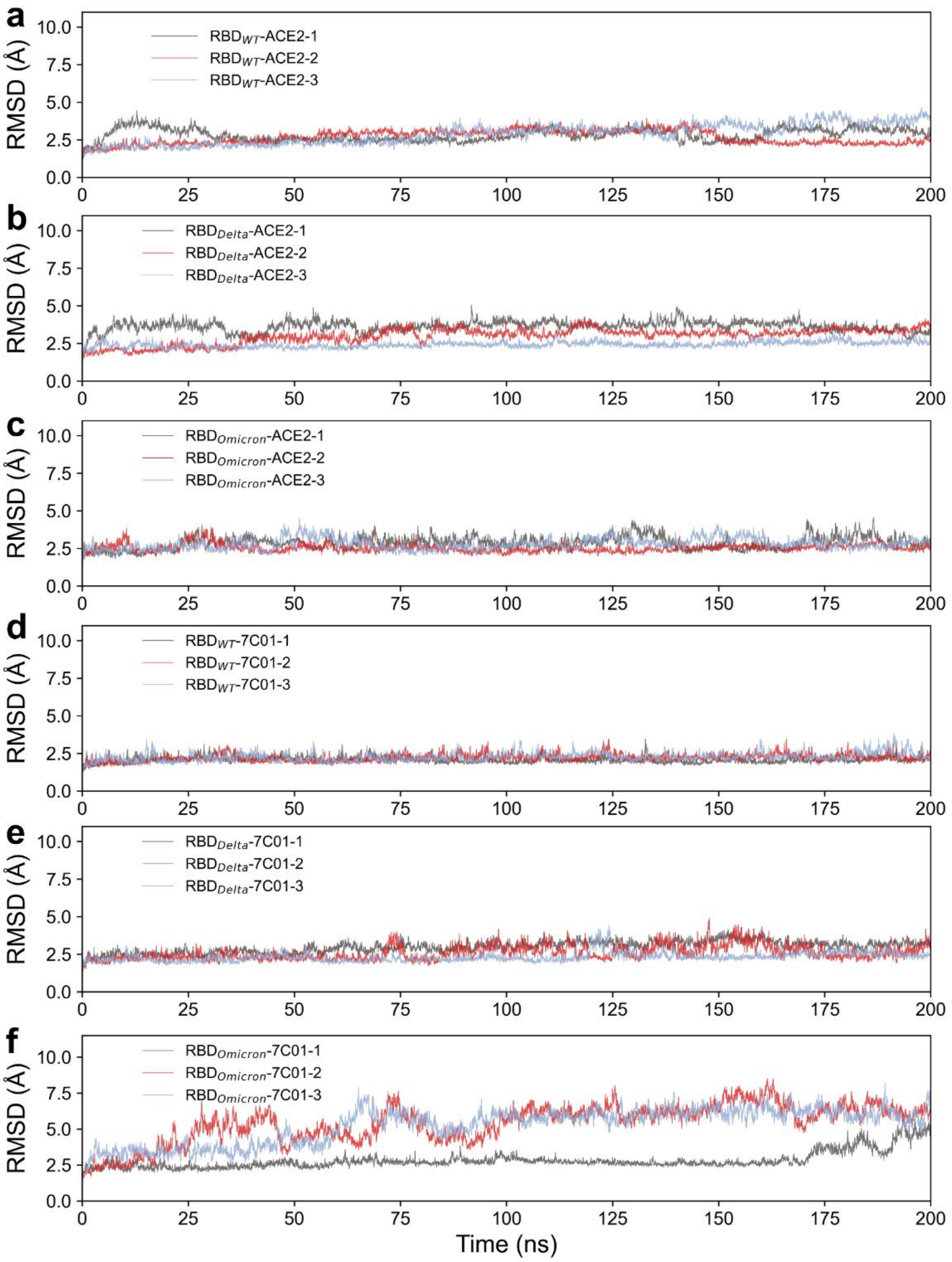

Figure S1. Time dependence of the heavy atom RMSD during 200 ns MD simulation. a, b and **c** for the systems formed by the different RBDs and ACE2. **d, e** and **f** for the systems formed by the different RBDs and Etesevimab (PDB ID: 7C01).

Figure S2

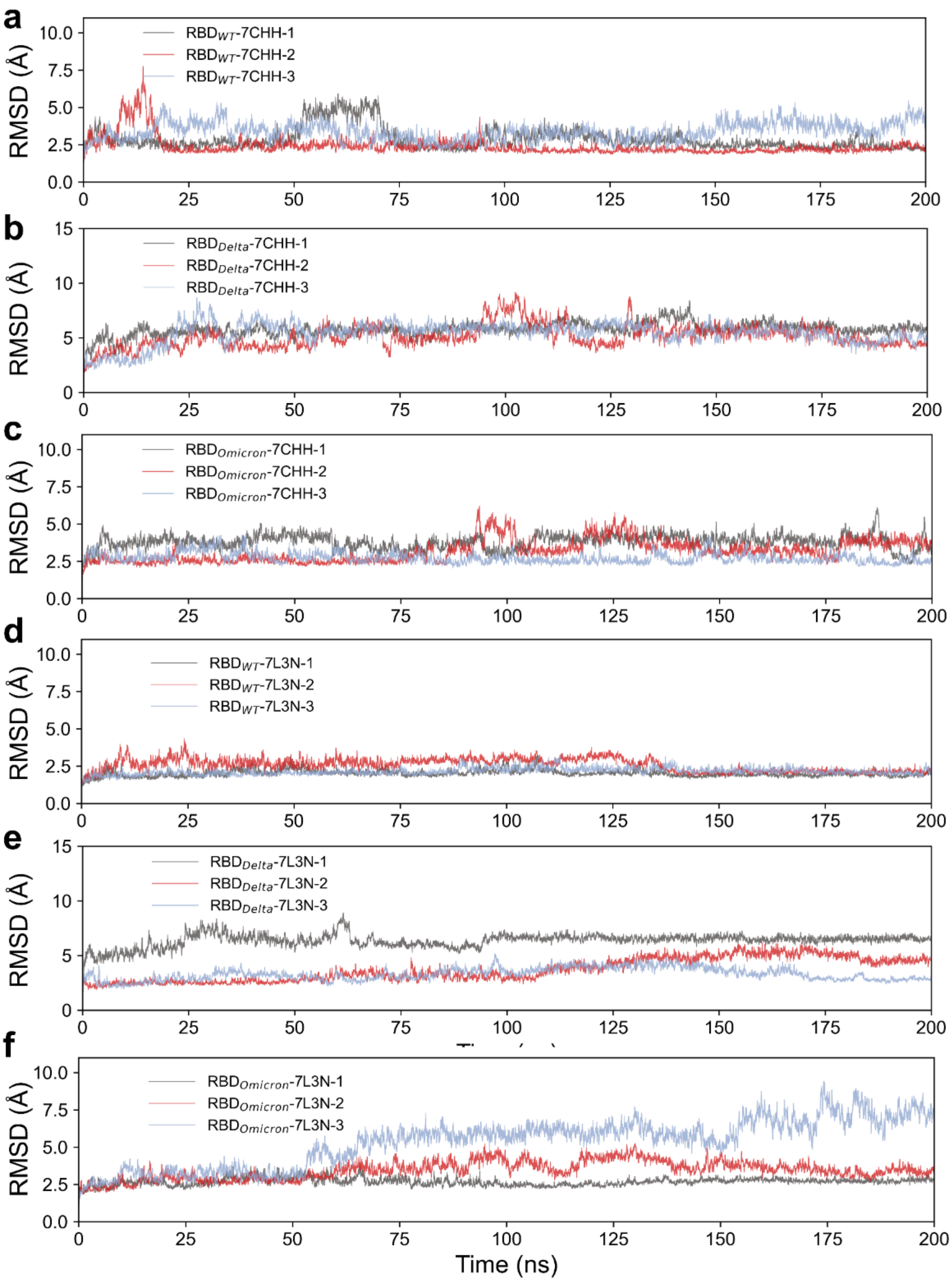

Figure S2. Time dependence of the heavy atom RMSD during 200 ns MD simulation. a, b and **c** for the systems formed by the different RBDs and BD-368-2 (PDB ID: 7CHH). **d, e** and **f** for the systems formed by the different RBDs and Bamlanivimab (PDB ID: 7L3N).

Figure S3

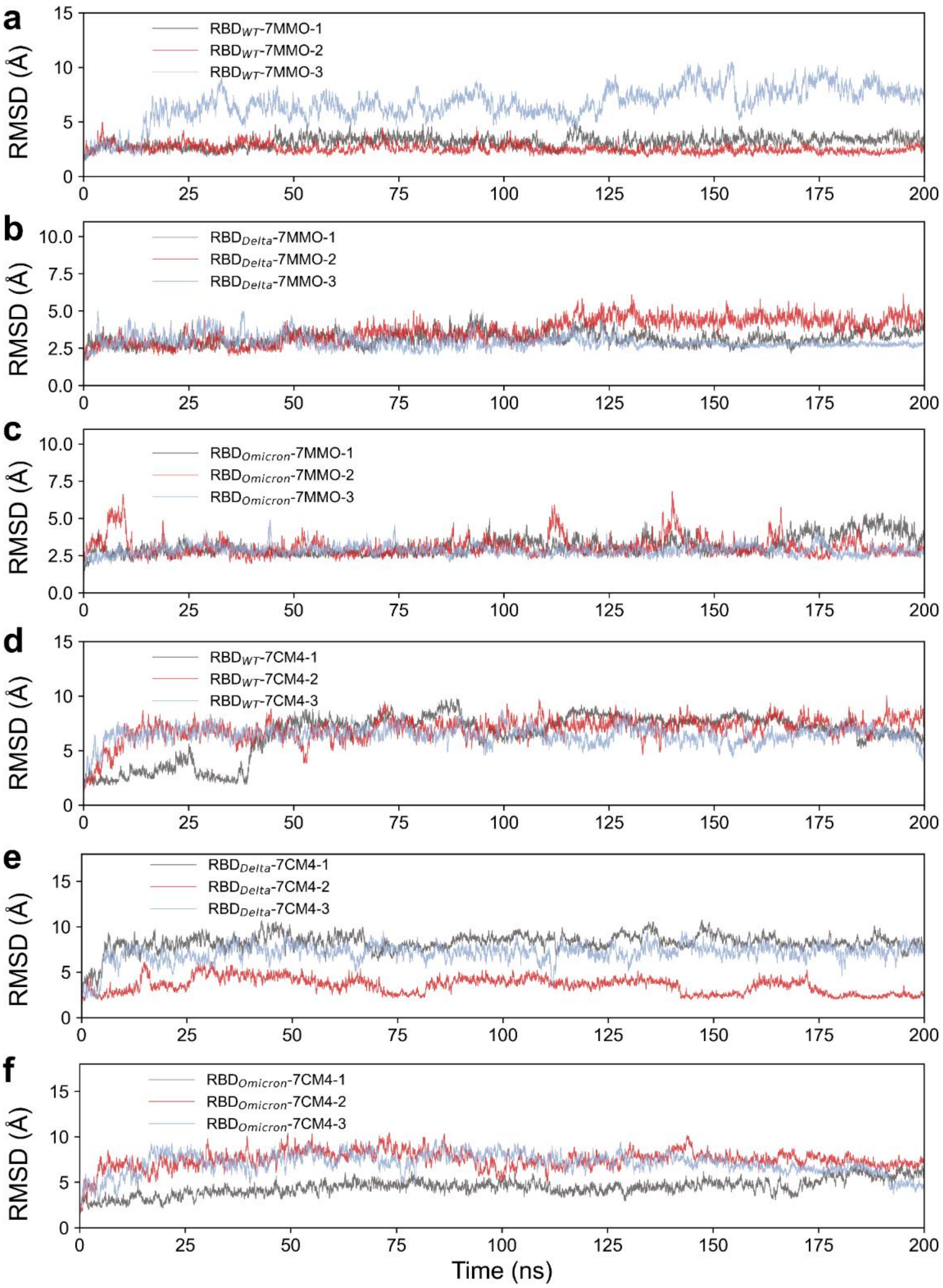

Figure S3. Time dependence of the heavy atom RMSD during 200 ns MD simulation. a, b and **c** for the systems formed by the different RBDs and Bebtelovimab (PDB ID: 7MMO). **d, e** and **f** for the systems formed by the different RBDs and Regdanvimab (PDB ID: 7CM4).

Figure S4

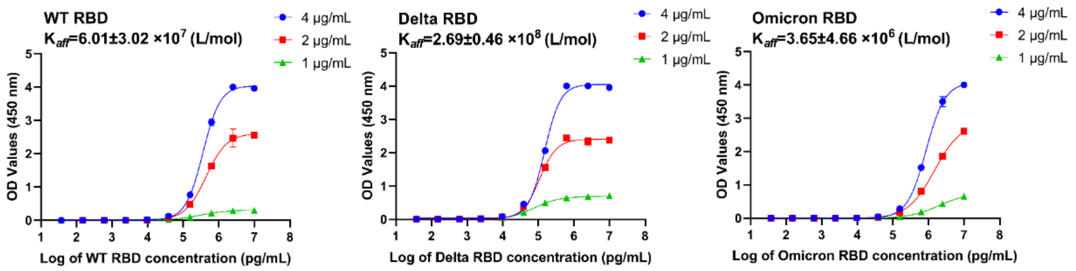

Figure S4. Experimental ELISA curves for three RBDs at different ACE2 coating concentrations. The calculated wild type RBD concentrations (ng/mL) at OD-50 were: 351.0 (4 μg/mL), 411.0 (2 μg/mL), and 287.0 (1 μg/mL), Delta RBD concentrations (ng/mL) at OD-50 were: 145.7 (4 μg/mL), 106.9 (2 μg/mL), and 77.4 (1 μg/mL), and Omicron RBD concentrations (ng/mL) at OD-50 were: 831.7 (4 μg/mL), 1246.2 (2 μg/mL), and 10146.6 (1 μg/mL).

Figure S5

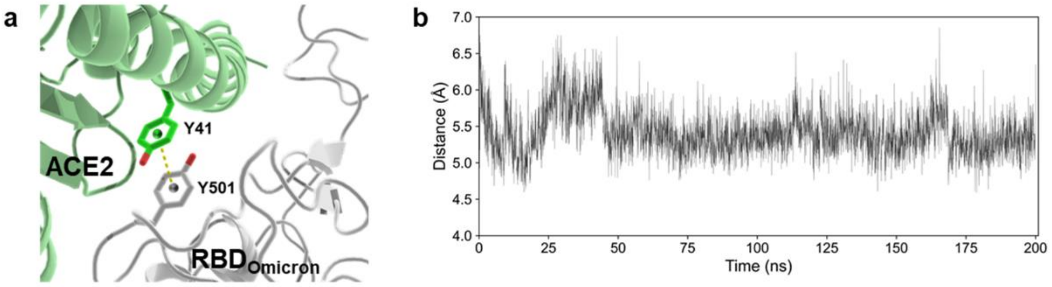

Figure S5. The interaction between $Y501_{RBD}$ and $Y41_{ACE2}$. (**a**) Diagram of $Y501_{RBD}$-$Y41_{ACE2}$ π-π stacking interaction. (**b**) The distance between the center of mass of $Y501_{RBD}$ and $Y41_{ACE2}$.

Figure S6

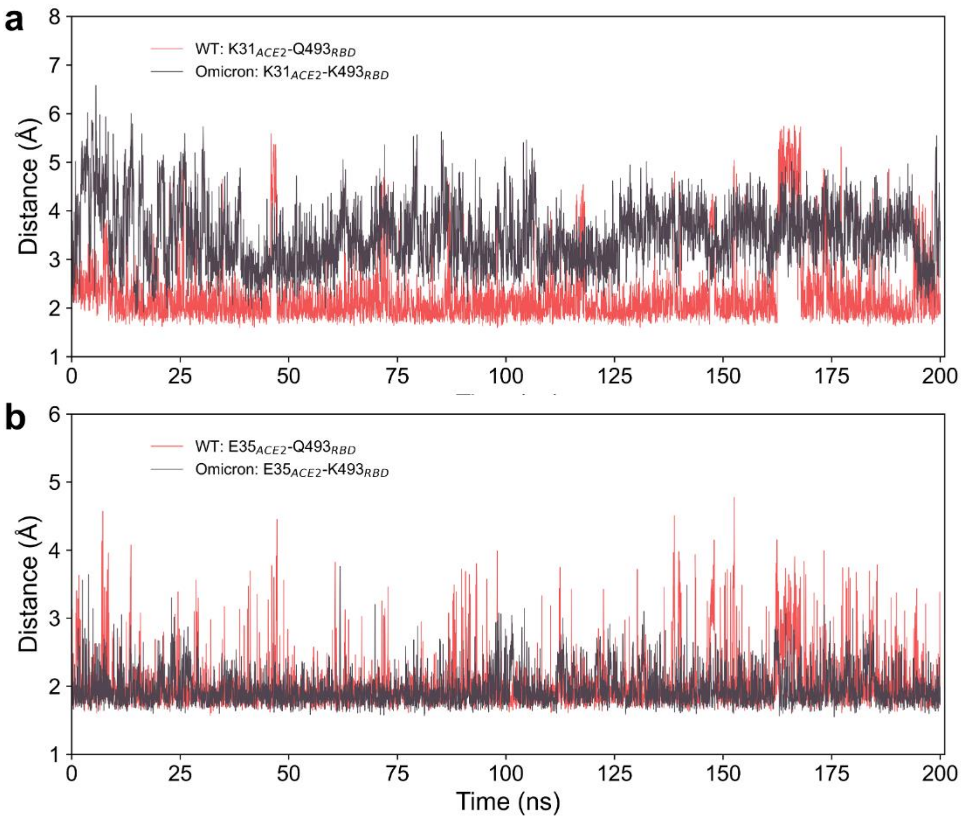

Figure S6. The dependence of minimum distance during 200 ns MD simulation. (**a**) Minimum distance between $K31_{ACE2}$ and $Q/K493_{RBD}$. (**b**) Minimum distance between $E35_{ACE2}$ and $Q/K493_{RBD}$.

Figure S7

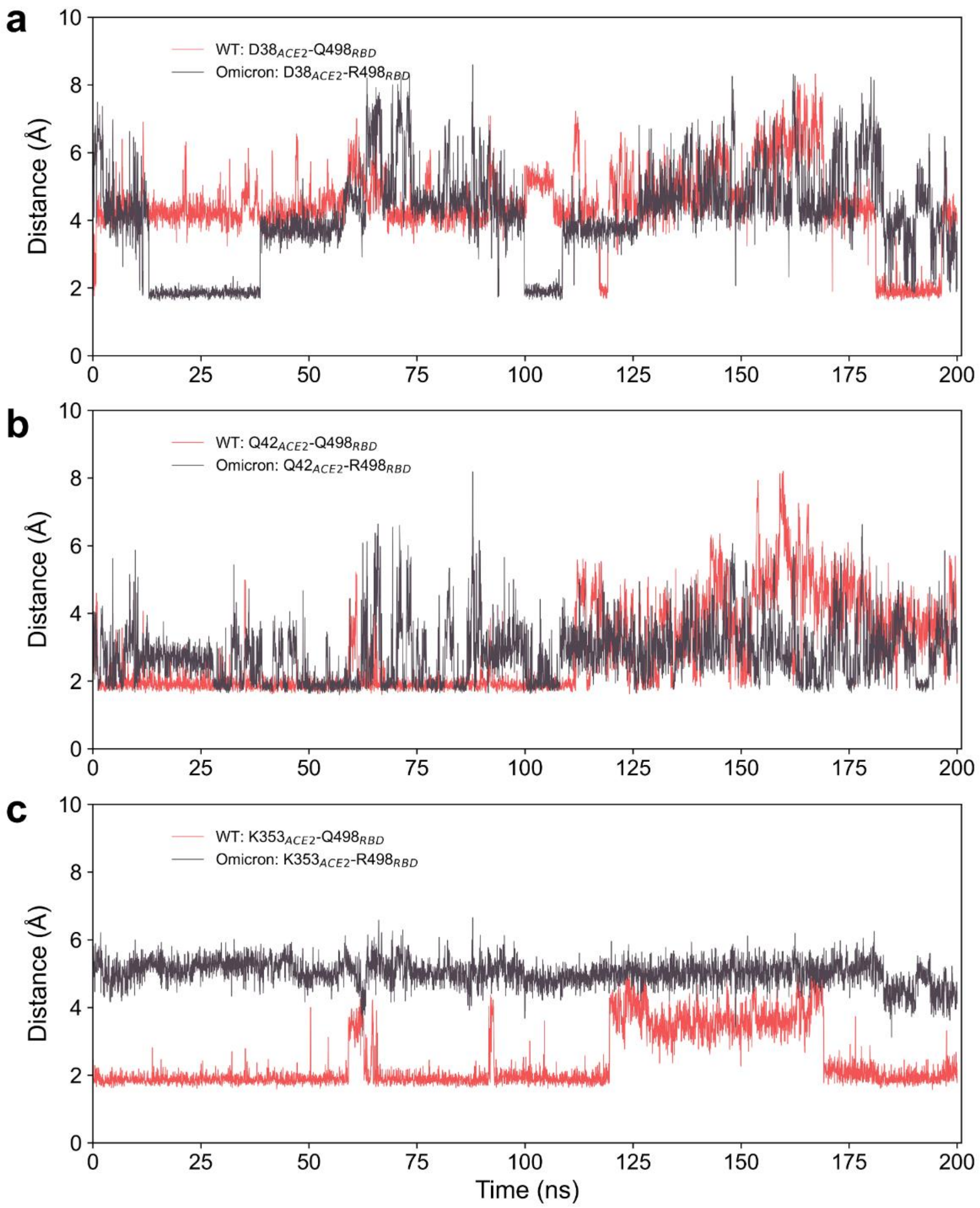

Figure S7. The dependence of minimum distance during 200 ns MD simulation (**a**) Minimum distance between D38$_{ACE2}$ and Q/R498$_{RBD}$. (**b**) Minimum distance between Q42$_{ACE2}$ and Q/R498$_{RBD}$. (**c**) Minimum distance between K353$_{ACE2}$ and Q/R498$_{RBD}$.

Figure S8

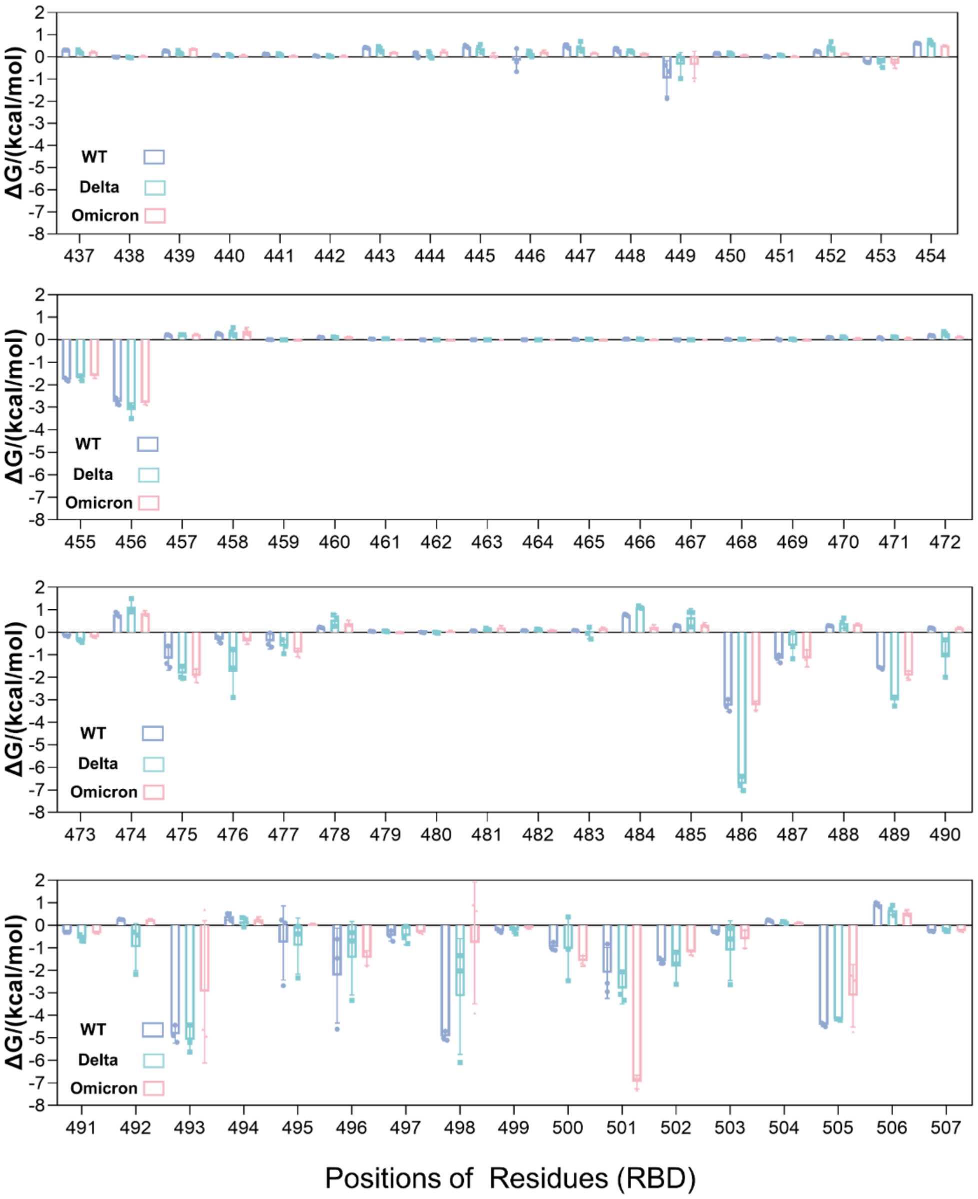

Figure S8. The binding free energy decomposition of ACE2 to RBM residues (437-507) in the different RBDs.

Table S1

Table S1. The initial pdb structures of the simulated systems.

Name (.pdb)	System	Original PDB ID
rbd_ace2_wt	RBD_{WT}-ACE2	6VW1
rbd_ace2_delta	RBD_{Delta}-ACE2	7VVS, 6VW1
rbd_ace2_omicron	$RBD_{Omicron}$-ACE2	7N1X, 6VW1
7c01_wt	RBD_{WT}-Etesevimab	7C01
7c01_delta	RBD_{Delta}-Etesevimab	7C01
7c01_omicron	$RBD_{Omicron}$-Etesevimab	7C01
7mmo_wt	RBD_{WT}-Bebtelovimab	7MMO
7mmo_delta	RBD_{Delta}-Bebtelovimab	7MMO
7mmo_omicron	$RBD_{Omicron}$-Bebtelovimab	7MMO
7cm4_wt	RBD_{WT}-Regdanvimab	7CM4
7cm4_delta	RBD_{Delta}-Regdanvimab	7CM4
7cm4_omicron	$RBD_{Omicron}$-Regdanvimab	7CM4
7chh_wt	RBD_{WT}-(BD-368-2)	7CHH
7chh_delta	RBD_{Delta}-(BD-368-2)	7CHH
7chh_omicron	$RBD_{Omicron}$-(BD-368-2)	7CHH
7l3n_wt	RBD_{WT}-Bamlanivimab	7L3N
7l3n_delta	RBD_{Delta}-Bamlanivimab	7L3N
7l3n_omicron	$RBD_{Omicron}$-Bamlanivimab	7L3N

Table S2

Table S2. The parameter files in gromacs format of the simulated systems.

System	PATH	Original PDB ID	File type
RBD$_{WT}$-ACE2	Data_S2\rbd_ace2\wt	6VW1	Topology, structure, index files in gromacs format
RBD$_{Delta}$-ACE2	Data_S2\rbd_ace2\delta	7VVS, 6VW1	
RBD$_{Omicron}$-ACE2	Data_S2\rbd_ace2\omicron	7N1X, 6VW1	
RBD$_{WT}$-Etesevimab	Data_S2\7c01\wt	7C01	
RBD$_{Delta}$-Etesevimab	Data_S2\7c01\delta	7C01	
RBD$_{Omicron}$-Etesevimab	Data_S2\7c01\omicron	7C01	
RBD$_{WT}$-Bebtelovimab	Data_S2\7mmo\wt	7MMO	
RBD$_{Delta}$-Bebtelovimab	Data_S2\7mmo\delta	7MMO	
RBD$_{Omicron}$-Bebtelovimab	Data_S2\7mmo\omicron	7MMO	
RBD$_{WT}$-Regdanvimab	Data_S2\7cm4\wt	7CM4	
RBD$_{Delta}$-Regdanvimab	Data_S2\7cm4\delta	7CM4	
RBD$_{Omicron}$-Regdanvimab	Data_S2\7cm4\omicron	7CM4	
RBD$_{WT}$-(BD-368-2)	Data_S2\7chh\wt	7CHH	
RBD$_{Delta}$-(BD-368-2)	Data_S2\7chh\delta	7CHH	
RBD$_{Omicron}$-(BD-368-2)	Data_S2\7chh\omicron	7CHH	
RBD$_{WT}$-Bamlanivimab	Data_S2\7l3n\wt	7L3N	
RBD$_{Delta}$-Bamlanivimab	Data_S2\7l3n\delta	7L3N	
RBD$_{Omicron}$-Bamlanivimab	Data_S2\7l3n\omicron	7L3N	

Table S3

Table S3. The predicted mAb-RBD binding free energy (kcal/mol) of WT, Delta and Omicron variants.

Antibody	PDB ID	System	Average	1	2	3
Etesevimab CB-6 (Launched)	7C01[24]	RBD_{WT}	-67.78 ± 2.12	-65.33 ± 0.34	-68.85 ± 0.33	-69.15 ± 0.34
		RBD_{Delta}	-42.34 ± 5.78	-37.64 ± 0.29	-40.6 ± 0.33	-48.79 ± 0.34
		$RBD_{Omicron}$	-39.75 ± 1.63	-36.28 ± 0.43	-38.04 ± 0.45	-39.54 ± 0.39
Bebtelovimab LY-CoV-1404 (Launched)	7MMO[25]	RBD_{WT}	-58.77 ± 2.97	-56.52 ± 0.32	-57.66 ± 0.40	-62.14 ± 0.58
		RBD_{Delta}	-53.96 ± 2.27	-51.49 ± 0.28	-54.45 ± 0.26	-55.95 ± 0.34
		$RBD_{Omicron}$	-59.09 ± 4.37	-54.76 ± 0.34	-59.02 ± 0.28	-63.50 ± 0.35
Regdanvimab CT-P59 (Launched)	7CM4[26]	RBD_{WT}	-48.86 ± 5.69	-43.94 ± 0.32	-47.55 ± 0.28	-55.10 ± 0.27
		RBD_{Delta}	-34.01 ± 10.39	-22.06 ± 0.33	-39.03 ± 0.33	-40.94 ± 0.37
		$RBD_{Omicron}$	-44.58 ± 4.39	-45.21 ± 0.53	-39.91 ± 0.32	-48.63 ± 0.34
BD-368-2 (Clinical)	7CHH[27]	RBD_{WT}	-28.30 ± 5.96	-22.42 ± 0.32	-28.14 ± 0.33	-34.33 ± 0.26
		RBD_{Delta}	-11.09 ± 5.86	-7.08 ± 0.24	-8.37 ± 0.26	-17.82 ± 0.33
		$RBD_{Omicron}$	-13.31 ± 6.81	-7.64 ± 0.21	-11.42 ± 0.40	-20.86 ± 0.29
Bamlanivimab LY-CoV-555 (Launched)	7L3N[28]	RBD_{WT}	-52.90 ± 0.29	-52.69 ± 0.24	-52.78 ± 0.25	-53.23 ± 0.27
		RBD_{Delta}	-21.84 ± 6.92	-17.43 ± 0.20	-18.28 ± 0.23	-29.81 ± 0.33
		$RBD_{Omicron}$	-22.42 ± 1.61	-20.78 ± 0.21	-22.49 ± 0.38	-23.99 ± 0.27

Movie S1.

The interaction modes between ACE2 and 3 different RBDs. The movie shows the molecular dynamics simulations of three RBDs (i.e. RBD in WT, Delta and Omicron) interacting with ACE2. For illustrative purposes, RBD is shown as blue cartoon, and ACE2 in transparent green cartoon. Mutations are labeled and shown as purple sticks on RBD_{Delta} and yellow sticks on $RBD_{Omicron}$. Only one 200ns MD trajectory was shown.

References

1 Waterhouse, A. *et al.* SWISS-MODEL: homology modelling of protein structures and complexes. *Nucleic Acids Res.* **46**, W296-W303 (2018).

2 Shang, J. *et al.* Structural basis of receptor recognition by SARS-CoV-2. *Nature.* **581**, 221-224 (2020).

3 Schrödinger, L. *The PyMOL Molecular Graphics System,Version 2.5* (2021).

4 Yang, T. J., Yu, P.Y., Chang, Y.C., Hsu, S.T.D. Cryo-EM structure of SARS-CoV-2 S-Delta variant (B.1.617.2), one RBD-up conformation 5. DOI: 10.2210/pdb7V7S/pdb (2021).

5 H++, http://biophysics.cs.vt.edu/H++, (accessed 1 December, 2021)..

6 Anandakrishnan, R., Aguilar, B. & Onufriev, A. V. H++ 3.0: automating pK prediction and the preparation of biomolecular structures for atomistic molecular modeling and simulations. *Nucleic Acids Res.* **40**, W537-541 (2012).

7 Duan, Y. *et al.* A point-charge force field for molecular mechanics simulations of proteins based on condensed-phase quantum mechanical calculations. *J. Comput. Chem.* **24**, 1999-2012 (2003).

8 Case D.A. *et al.* AMBER 2018, University of California, San Francisco (2018).

9 Abraham, M. J. *et al.* GROMACS: High performance molecular simulations through multi-level parallelism from laptops to supercomputers. *SoftwareX* **1-2**, 19-25 (2015).

10 Lindahl, A., Hess & van der Spoel. GROMACS 2020.2 Manual (2020.2). Zenodo., https://doi.org/10.5281/zenodo.3773799 (accessed 1 December, 2021).

11 Ermak, D. L. & McCammon, J. A. Brownian dynamics with hydrodynamic interactions. *The Journal of Chemical Physics* **69**, 1352-1360 (1978).

12 Feller, S. E., Zhang, Y., Pastor, R. W. & Brooks, B. R. Constant pressure molecular dynamics simulation: The Langevin piston method. *The Journal of Chemical Physics* **103**, 4613-4621 (1995).

13 Martyna, G. J., Tobias, D. J. & Klein, M. L. Constant pressure molecular dynamics algorithms. *The Journal of Chemical Physics* **101**, 4177-4189 (1994).

14 Ryckaert, J.-P., Ciccotti, G. & Berendsen, H. J. C. Numerical integration of the

cartesian equations of motion of a system with constraints: molecular dynamics of n-alkanes. *Journal of Computational Physics* **23**, 327-341 (1977).

15 Essmann, U. *et al.* A smooth particle mesh Ewald method. *The Journal of Chemical Physics* **103**, 8577-8593 (1995).

16 McGibbon, R. T. *et al.* MDTraj: A Modern Open Library for the Analysis of Molecular Dynamics Trajectories. *Biophys J* **109**, 1528-1532 (2015).

17 Kollman, P. A. *et al.* Calculating structures and free energies of complex molecules: combining molecular mechanics and continuum models. *Acc. Chem. Res.* **33**, 889-897 (2000).

18 Beatty, J. D., Beatty, B. G. & Vlahos, W. G. Measurement of monoclonal antibody affinity by non-competitive enzyme immunoassay. *J. Immunol. Methods* **100**, 173-179 (1987).

19 Liu, H. *et al.* The Lambda variant of SARS-CoV-2 has a better chance than the Delta variant to escape vaccines. Preprint at https://www.biorxiv.org/content/10.1101/2021.08.25.457692v1 (2021).

20 Augusto, G. *et al.* In vitro data suggest that Indian delta variant B.1.617 of SARS-CoV-2 escapes neutralization by both receptor affinity and immune evasion. *Allergy*, https://doi.org/10.1111/all.15065 (2021).

21 Cameroni, E. *et al.* Broadly neutralizing antibodies overcome SARS-CoV-2 Omicron antigenic shift. Preprint at https://www.biorxiv.org/content/10.1101/2021.12.12.472269v1 (2021).

22 Cao, Y. *et al.* B.1.1.529 escapes the majority of SARS-CoV-2 neutralizing antibodies of diverse epitopes. Preprint at https://www.biorxiv.org/content/10.1101/2021.12.07.470392v1 (2021).

23 Liu, L. *et al.* Striking Antibody Evasion Manifested by the Omicron Variant of SARS-CoV-2. Preprint at https://www.biorxiv.org/content/10.1101/2021.12.14.472719v1 (2021).

24 Shi, R. *et al.* A human neutralizing antibody targets the receptor-binding site of SARS-CoV-2. *Nature.* **584**, 120-124 (2020).

25 Westendorf, K. *et al.* LY-CoV1404 potently neutralizes SARS-CoV-2 variants. Preprint

at https://www.biorxiv.org/content/10.1101/2021.04.30.442182v4 (2021).

26 Kim, C. *et al.* A therapeutic neutralizing antibody targeting receptor binding domain of SARS-CoV-2 spike protein. *Nat. Commun.* **12**, 288 (2021).

27 Du, S. *et al.* Structurally Resolved SARS-CoV-2 Antibody Shows High Efficacy in Severely Infected Hamsters and Provides a Potent Cocktail Pairing Strategy. *Cell.* **183**, 1013-1023.e1013 (2020).

28 Jones, B. E. *et al.* LY-CoV555, a rapidly isolated potent neutralizing antibody, provides protection in a non-human primate model of SARS-CoV-2 infection. Preprint at https://www.biorxiv.org/content/10.1101/2020.09.30.318972v3 (2020).
