# Supplementary figures and images for: SARS-CoV-2 Omicron RBD shows weaker binding affinity than the currently dominant Delta variant to human ACE2
Source: Signal Transduct Target Ther. 2022 Jan 5;7:8. doi: 10.1038/s41392-021-00863-2 (PMC8727475; doi:10.1038/s41392-021-00863-2)

## Slide 1
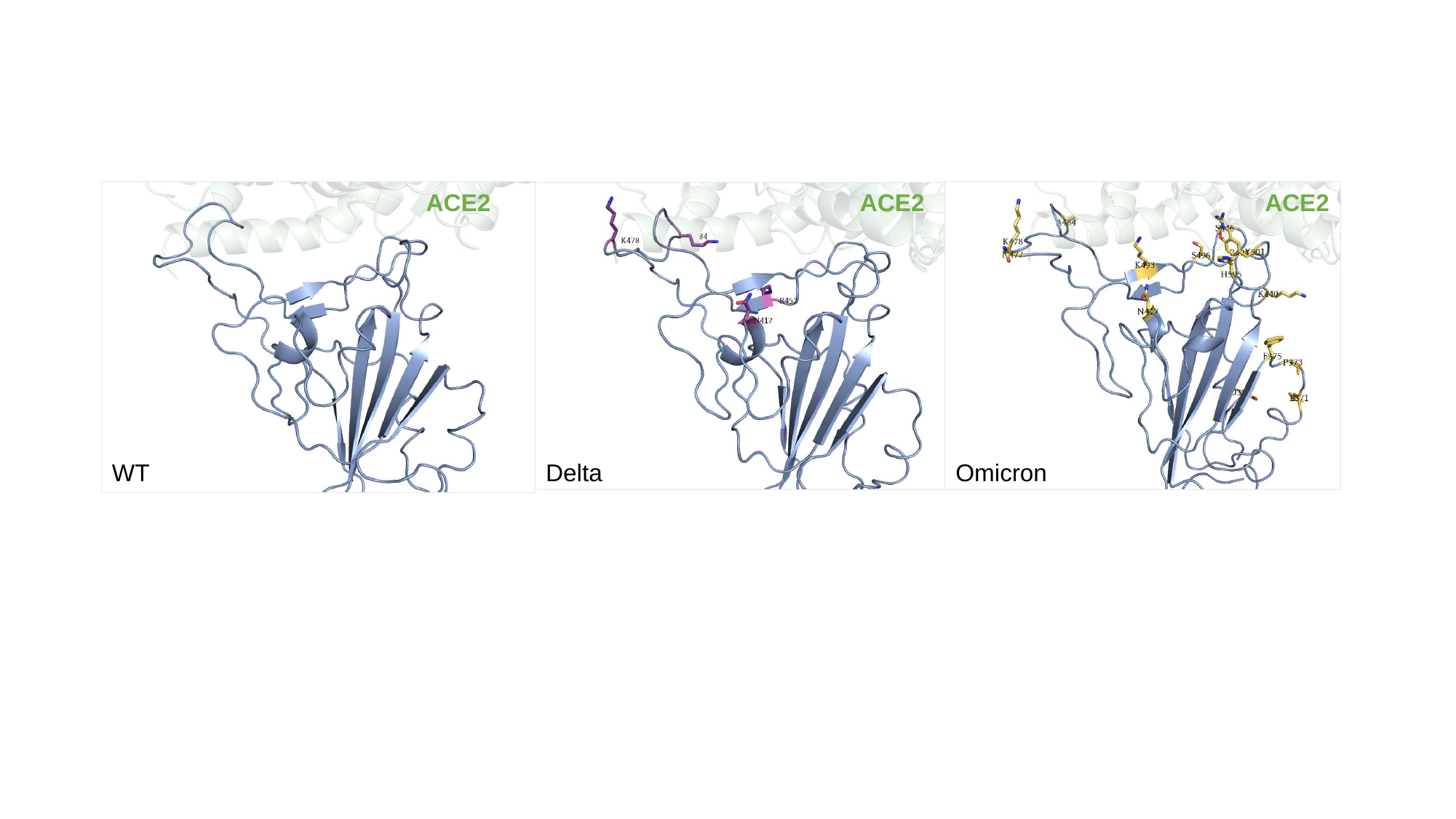

ACE2
ACE2
ACE2
WT
Delta
Omicron

Supplement: Supplementary file 2 — Movie S1 The interaction modes between ACE2 and 3 different RBDs [file 41392_2021_863_MOESM2_ESM.pptx]
